# Immunostimulatory Activity of *Syneilesis palmata* Leaves through Macrophage Activation and Macrophage Autophagy in Mouse Macrophages, RAW264.7 Cells

**DOI:** 10.4014/jmb.2301.01039

**Published:** 2023-04-19

**Authors:** So Jung Park, Jeong Won Choi, Hyeok Jin Choi, Seung Woo Im, Jin Boo Jeong

**Affiliations:** Department of Forest Science, Andong National University, Andong 36729, Republic of Korea

**Keywords:** Immunostimulatory activity, macrophage activation, macrophage autophagy, *Syneilesis palmata*

## Abstract

*Syneilesis palmata* (SP) is a traditional medicinal plant. SP has been reported to have anti-inflammatory, anticancer, and anti-human immunodeficiency virus (HIV) activities. However, there is currently no research available on the immunostimulatory activity of SP. Therefore, in this study, we report that *S. palmata* leaves (SPL) activate macrophages. Increased secretion of both immunostimulatory mediators and phagocytic activity was observed in SPL-treated RAW264.7 cells. However, this effect was reversed by the inhibition of TLR2/4. In addition, inhibition of p38 decreased the secretion of immunostimulatory mediators induced by SPL, and inhibition of TLR2/4 decreased the phosphorylation of p38 induced by SPL. SPL augmented p62/SQSTM1 and LC3-II expression. The increase in protein levels of p62/SQSTM1 and LC3-II induced by SPL was decreased by the inhibition of TLR2/4. The results obtained from this study suggest that SPL activates macrophages via TLR2/4-dependent p38 activation and induces autophagy in macrophages via TLR2/4 stimulation.

## Introduction

The ongoing COVID-19 pandemic has increased the interest in boosting immunity. Enhancing immunity can decrease the fatality rate and mitigate long-term impairments resulting from COVID-19 [1]. Enhancing innate immunity is reported as a crucial defense mechanism against COVID-19, as it can improve resistance to COVID-19 and prevent various diseases caused by it [2].

It is known that vegetable-based diets can enhance the immune system's ability to protect the body against foreign pathogens [3]. *Syneilesis palmata*, belonging to the Asteraceae family, has been traditionally used as both a medicinal herb and vegetable for a long time in Korea. has been traditionally used as a medicinal herb primarily for treating inflammatory disorders and improving blood circulation [4, 5]. Various pharmacological studies have revealed that *Syneilesis palmata* exhibits anti-inflammatory [4], anticancer [5, 6], and anti-human immunodeficiency virus type 1 (HIV-1) [7] activities. However, there have been no studies on the immune-enhancing effects of *Syneilesis palmata* so far. Therefore, this study reports that *Syneilesis palmata* activates macrophages, which are innate immune cells, as evidence of *Syneilesis palmata* 's immune-boosting activity.

## Materials and Methods

### Materials

MTT (3-(4,5-dimethylthiazol-2-yl)-2,5-diphenyl-2H-tetrazolium bromide), neutral red, Griess, and chemical inhibitors (TAK-242, C29, PD98059, SB203580, and SP600125) were purchased from Sigma-Aldrich (USA). Primary antibodies (p-p38, LC3, p62/SQSTM1, and *β*-actin) and secondary antibody (anti-rabbit) were purchased from Cell Signaling Technology (USA). RNeasy Mini Kit was purchased from Qiagen (Germany). Verso cDNA kit was purchased from Thermo Scientific (USA). PCR Master Mix Kit was purchased from Promega (USA).

### *Syneilesis palmata* Leaf (SPL) Extraction

*Syneilesis palmata* leaf was obtained from the National Institute of Forest Science (Korea). The crushed *S. palmata* leaf was immersed in 100% distilled water or 30-70% ethanol at 20 times the volume and extracted at 20-60°C for 1-24 h. Then, the clear extracts were freeze-dried. The freeze-dried *Syneilesis palmata* leaf extract (SPL) was dissolved in sterile water and used for this study. The absence of endotoxins in the SPL was verified using a Plant Endotoxin (EDT) enzyme-linked immunosorbent assay (ELISA) Kit (USA).

### Cell Culture

RAW264.7 cells (American Type Culture Collection, USA) were grown in Dulbecco’s modified Eagle medium (DMEM) F-12 supplemented with 10% fetal bovine serum (FBS) and maintained in a CO_2_ incubator (5% CO_2_, 37°C, humidified atmosphere).

### MTT Assay

To evaluate the cytotoxic effect of SPL on RAW264.7 cells, an MTT assay was performed. Briefly, RAW264.7 cells were treated with SPL for 24 h. Then, MTT solution was added to RAW264.7 cells (5% CO_2_, 37°C, humidified atmosphere) for an additional 4 h. Then, after dissolving the purple formazan from RAW264.7 cells with dimethyl sulfoxide (DMSO), the absorbance was analyzed at 570 nm using a UV/Visible spectrophotometer (Human Cop., Xma-3000PC, Korea).

### Griess Assay

The effect of SPL on NO production in RAW264.7 cells was analyzed using the Griess assay. After treating RAW264.7 cells with SPL and incubating them for 24 h, the cell culture supernatant was mixed with Griess reagent in a 1:1 ratio and incubated at room temperature for 15 min. Absorbance was measured at 540 nm using a UV/Visible spectrophotometer (Human Cop., Xma-3000PC).

### Neutral Red (NR) Assay

Neutral Red (NR) is commonly used to confirm phagocytic activity because it stains the lysosomes, which are essential for phagocytosis [8]. The effect of SPL on the phagocytic activity of RAW264.7 cells was analyzed using the NR assay. Briefly, RAW264.7 cells were treated with SPL and incubated for 24 h. Then, the cells were stained with 0.01% NR in an incubator (5% CO_2_, 37°C, humidified atmosphere) for 2 h. Next, after dissolving NR stained with RAW264.7 cells with lysis buffer (50% EtOH:1% acetic acid = 1:1), the absorbance was analyzed at 540 nm using a UV/Visible spectrophotometer (Human Cop., Xma-3000PC).

### Western Blot Analysis

Quantitative analysis of cellular proteins was performed using a bicinchoninic acid (BCA) assay kit (Thermo Fisher Scientific). Proteins were separated using sodium dodecyl sulfate-polyacrylamide gel electrophoresis and transferred to a nitrocellulose membrane (Thermo Fisher Scientific). The membranes were blocked for 1 h at room temperature and treated with primary antibodies (p-p38, LC3, p62/SQSTM1, or *β*-actin) at 4°C overnight. The membranes were then incubated with a secondary antibody (anti-rabbit) at room temperature for 1 h. After treating the membrane with ECL western blotting substrate, protein bands were visualized using an (Li-COR Biosciences, USA).

### Reverse Transcription-Polymerase Chain Reaction

mRNA of RAW264.7 cells were isolated using an RNeasy Mini Kit. mRNA isolated from RAW264.7 cells was quantified using a GeneQuant 1300 (Biochrom, England). cDNA was prepared from reverse transcription of mRNA (1 μg) using a (Thermo Scientific, USA). The cDNA was amplified using a (Promega, USA) and primers. The primer sequences used for cDNA amplification are shown in Table 1.

### Statistical Analysis

All data are presented as mean ± standard deviation (SD). Statistical significance was determined using Student’s *t*-test (Microsoft Excel 2013). *p < 0.05 and #*p* < 0.05 were considered statistically significant.

## Results

### SPL Extraction Conditions for High-Efficiency Immunostimulatory Activity

**Fig. 1:**
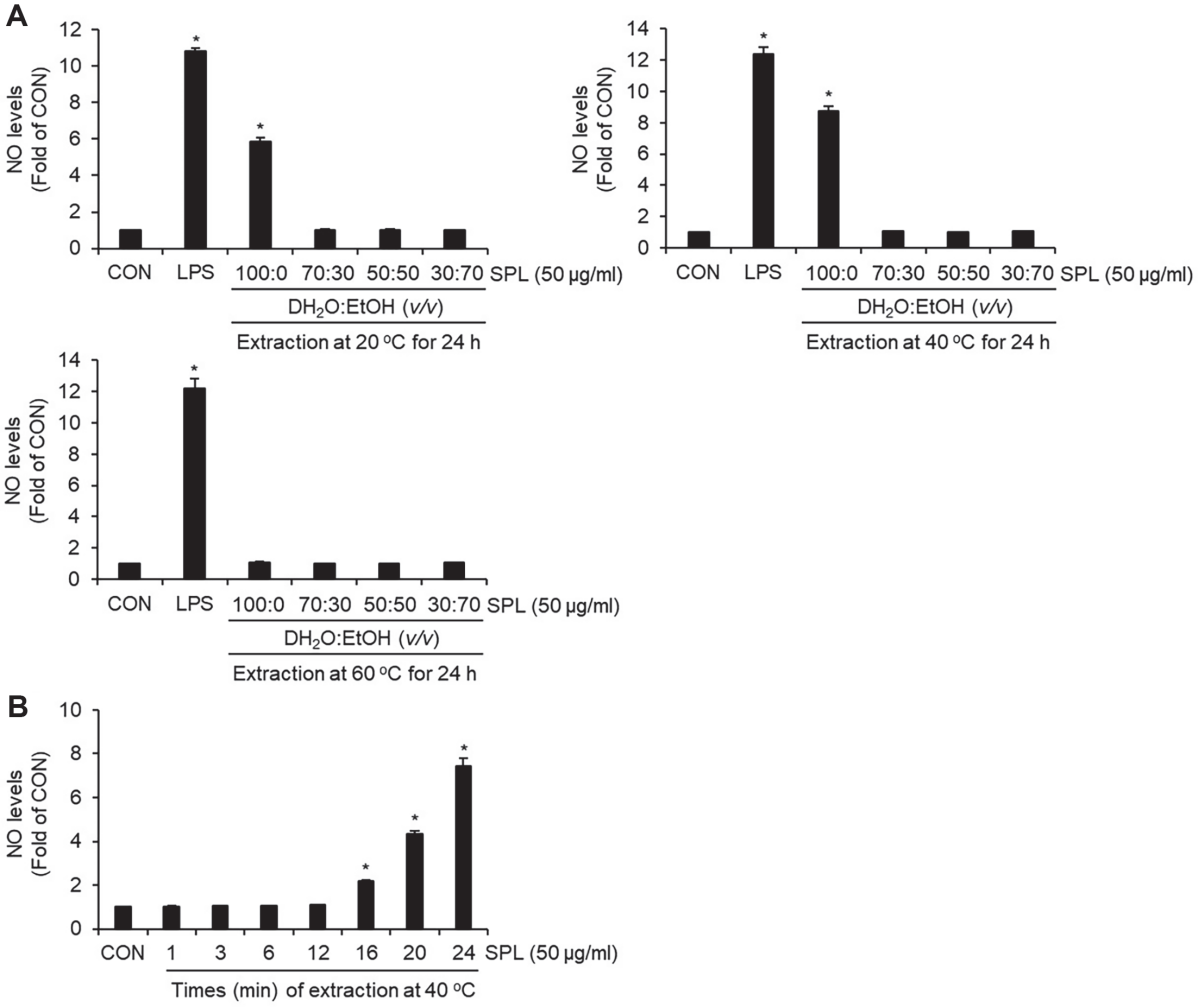
Effect of SPL on NO production according to extraction conditions in RAW264.7 cells. (**A**) SPL was extracted with 100% distilled water, 30% ethanol, 50% ethanol, or 70% ethanol at 20°C, 40°C, or 60°C for 24 h. (**B**) SPL was extracted with 100% distilled water at 40 ° for 1~24 h. NO level was measured by Griess assay.

We compared NO production by SPL under different conditions in RAW264.7 cells to determine the extraction conditions for the high-efficiency immunostimulatory activity of SPL. As shown in Fig. 1A, NO production was increased only in cells treated with SPL extracted with 100% distilled water and not in those treated with 30 to 70%ethanol-extracted SPL. However, SPL extracted with 100% distilled water at 20 and 40°C increased NO production in an upward direction, but SPL extracted at 60°C did not increase NO production. Thus, we used SPL extracted with 100% distilled water at 40°C and compared NO production in RAW264.7 cells at different conditions. As shown in Fig. 1B, NO production was not induced in SPL extracted for 1–12 h, but was increased in SPL extracted for 16–24 h. Therefore, we used SPL extracted with 100% distilled water at 40°C for 24 h in all subsequent experiments.

### Effect of SPL on the Activation of RAW264.7 Cells

**Fig. 2:**
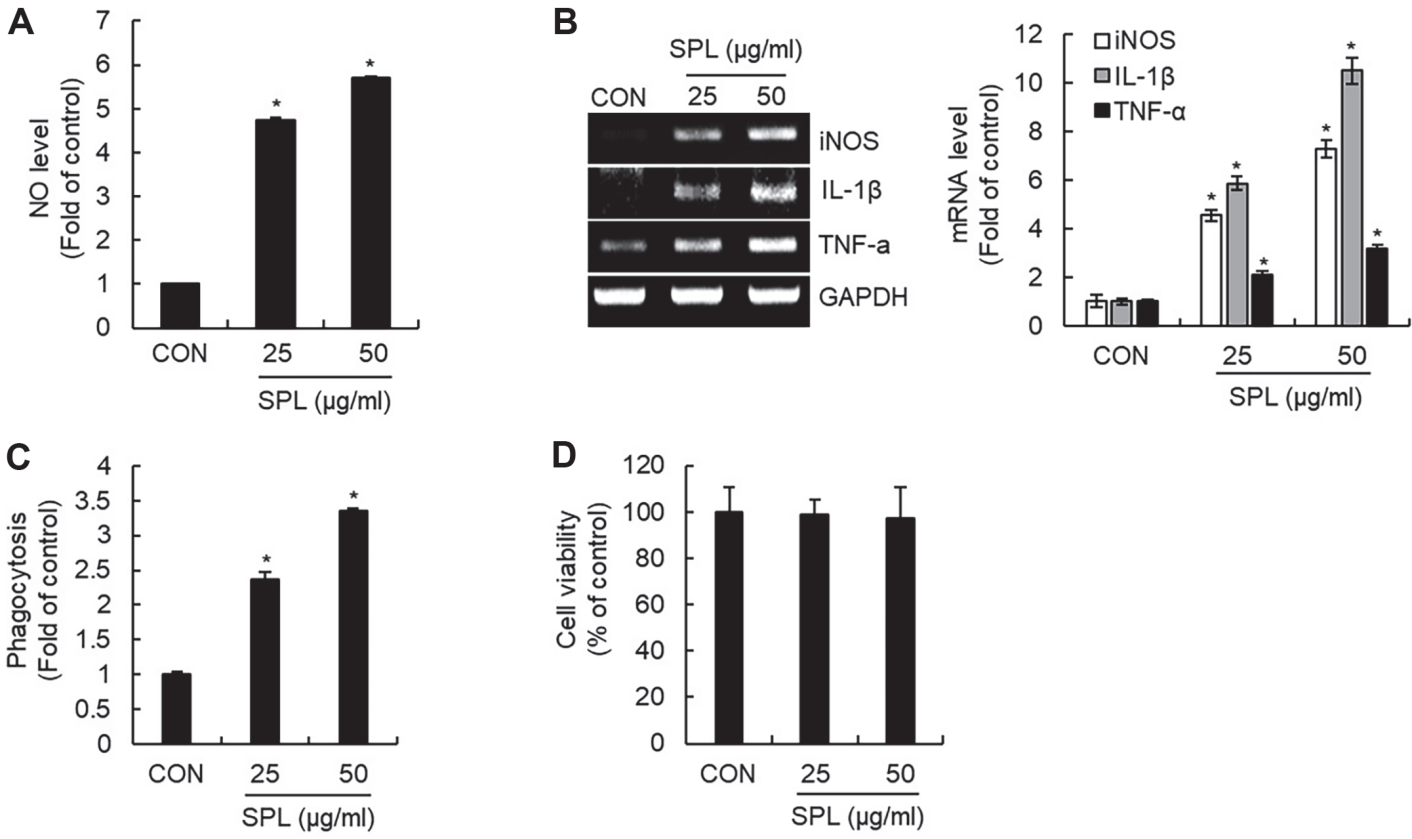
Effect of SPL on macrophage activation in RAW264.7 cells. RAW264.7 cells were treated with SPL for 24 h. NO level (**A**) mRNA level (**B**) phagocytotic activity (**C**), and cell viability (**D**) were measured by Griess assay, RT-PCR, neutral red assay, and MTT assay, respectively.

We measured the level changes of immunostimulatory mediators such as NO, inducible nitric oxide synthase (iNOS), interleukin-1β (IL-1β), and tumor necrosis factor-α (TNF-α) in SPL-treated RAW264.7 cells to investigate whether SPL affects the activation of RAW264.7 cells. SPL treatment increased the levels of NO, iNOS, IL-1β, and TNF-α (Figs. 2A and 2B). Since the upregulation of these mediators signifies macrophage activation, we investigated whether SPL enhances phagocytic activity, which serves as a functional marker of macrophage activation. The phagocytotic activity was promoted in SPL-treated RAW264.7 cells (Fig. 2C). However, SPL did not exhibit any cytotoxicity towards RAW264.7 cells (Fig. 2D).

### Effects of TLR2/4 on the Production of SPL-Mediated Immunostimulatory Mediators in RAW264.7 Cells

**Fig. 3:**
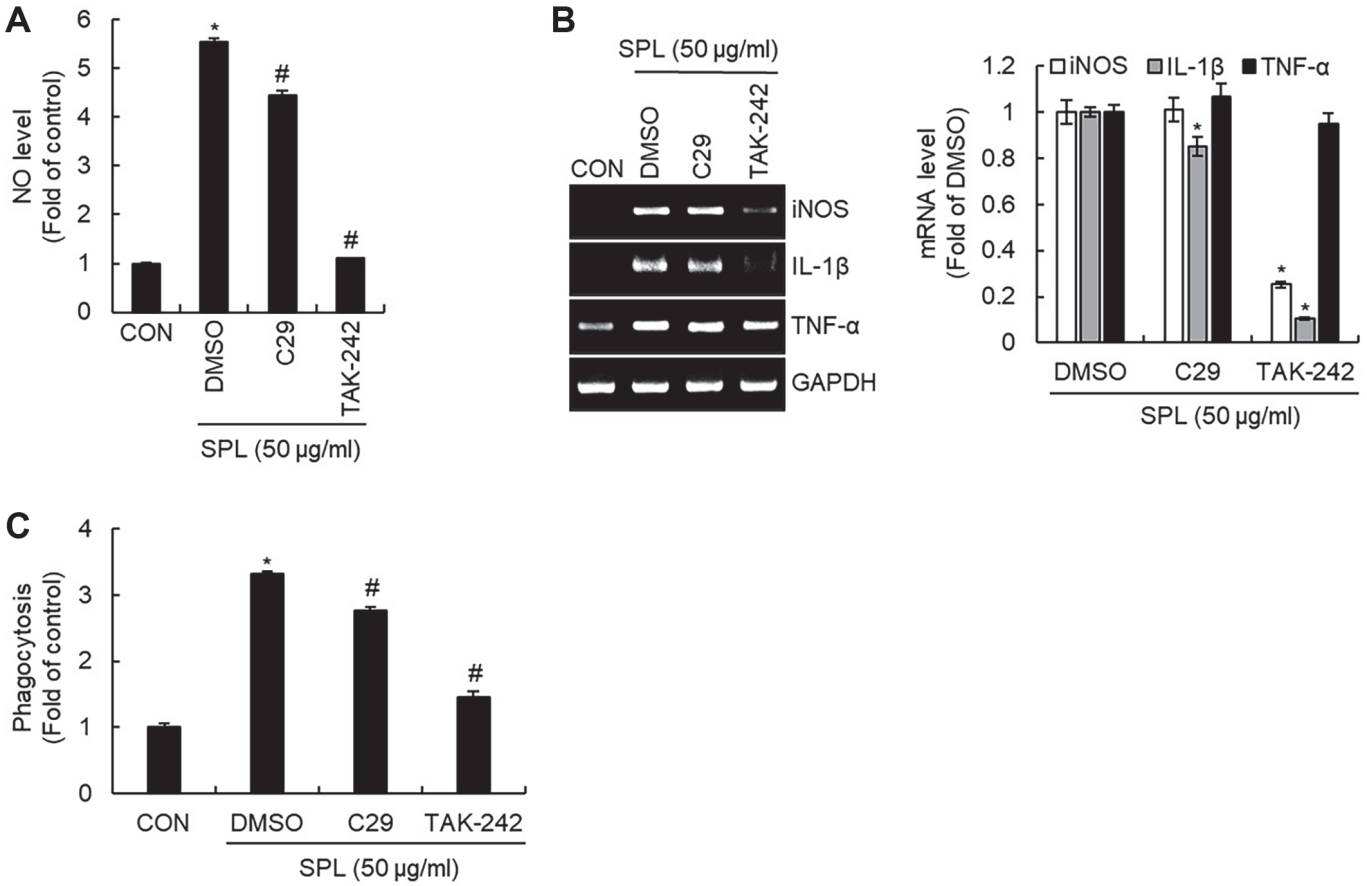
Effect of TLR2/4 on SPL-mediated macrophage activation in RAW264.7 cells. RAW264.7 cells were pretreated with C29 (TLR2 inhibitor, 100 μM) or TAK-242 (TLR4 inhibitor, 5 μM) for 2 h and co-treated with SPL for 24 h. NO level (**A**) and mRNA level (**B**) and phagocytotic activity (**C**) were measured by Griess assay, RT-PCR, and neutral red assay, respectively.

We investigated the effects of TLR2/4 on the secretion of immunostimulatory mediators and phagocytosis induced by SLP. As shown in Figs. 3A and 3B, the production of SPL-induced NO and IL-1β was slightly decreased in TLR2-inhibited RAW264.7 cells by C29. However, TAK-242-mediated inhibition of TLR4 significantly attenuated the SPL-mediated increases in the production of NO, iNOS, and IL-1β. Interestingly, inhibition of TLR2/4 did not affect SPL-mediated TNF-α production. In addition, the phagocytic activity of RAW264.7 cells induced by SPL was weakly reduced by TLR2 inhibition but significantly decreased by TLR4 inhibition (Fig. 3C).

### The Effect of Mitogen-Activated Protein Kinases (MAPKs) on the Production of Immunostimulatory Mediators Mediated by SPL in RAW264.7 Cells

**Fig. 4:**
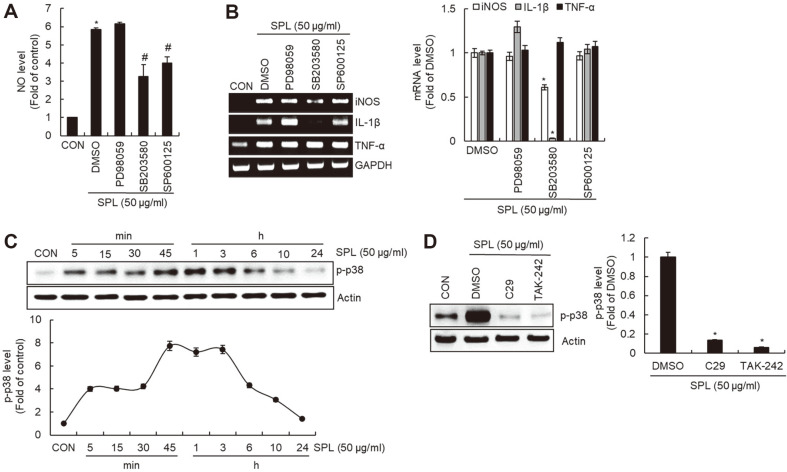
Effect of MAPK signaling pathway on SPL-mediated production of immunostimulatory mediators in RAW264.7 cells. RAW264.7 cells were pretreated with PD98059 (ERK1/2 inhibitor, 40 μM), SB203580 (p38 inhibitor, 40 μM), or SP600125 (JNK inhibitor, 40 μM) for 2 h and then co-treated with SPL for 24 h. NO level (**A**) and mRNA level (**B**) were measured by Griess assay and RT-PCR, respectively. (**C**) RAW264.7 cells were treated with SPL for the indicated times. The protein levels were determined by Western blot analysis. Actin was used as a loading control. (**D**) RAW264.7 cells were pretreated with C29 (TLR2 inhibitor, 100 μM) or TAK-242 (TLR4 inhibitor, 5 μM) for 2 h and co-treated with SPL for 1 h. Protein levels were measured by Western blot analysis. Actin was used as a loading control.

We investigated the effect of extracellular signal-regulated protein kinase 1/2 (ERK1/2), p38, or c-Jun N-terminal kinase (JNK) on the SPL-mediated secretion of immunostimulatory mediators. As shown in Figs. 4A and 4B, the inhibition of ERK1/2 by PD98059 did not affect the production of NO, iNOS, IL-1β, and TNF-α induced by SPL. Inhibition of p38 by SB203580 significantly attenuated the SPL-mediated production of NO, iNOS, and IL-1β, but had no effect on TNF-α production. SP600125-mediated inhibition of JNK inhibited SPL-mediated NO production. These results indicate that p38 is a major signaling pathway involved in the secretion of immunostimulatory mediators by SPL. Thus, we evaluated the effect of SPL on p38 activation and that of TLR2/4 on SPL-mediated p38 activation. Phosphorylation of p38 increased 5 min after SPL treatment, reached its maximum at 1 h, and then gradually decreased (Fig. 4C), indicating that SPL activates p38. In addition, C29-mediated inhibition of TLR2 and TAK-242-mediated inhibition of TLR4 decreased SPL-mediated p38 phosphorylation (Fig. 4D).

### Effect of SPL on RAW264.7 Cell Autophagy

**Fig. 5:**
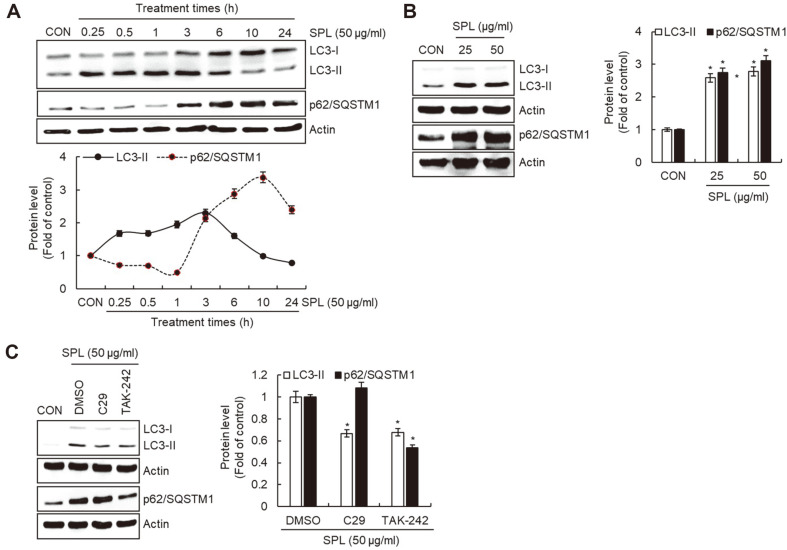
Effect of SPL on macrophage autophagy. (**A**) RAW264.7 cells were treated with SPL for the indicated times. (**B**) RAW264.7 cells were treated with 25 or 50 ul/ml of SPL. (**C**) RAW264.7 cells were pretreated with C29 (TLR2 inhibitor, 100 μM) or TAK-242 (TLR4 inhibitor, 5 μM) for 2 h and then co-treated with SPL for 1 h or 6 h. The protein levels were determined by Western blot analysis. Actin was used as a loading control. The protein levels were determined by Western blot analysis. Actin was used as a loading control.

To investigate the effect of SPL on autophagy in macrophages, we measured the protein levels of microtubule-associated protein 1A/1B-light chain 3-II (LC3-II) and p62/SQSTM1 in SPL-treated RAW264.7 cells using Western blot analysis. As shown in Fig. 5A, the protein level of LC3-II began to increase at 15 min after SPL treatment and reached its maximum at 3 h after SPL treatment, and then started to decrease. The protein level of p62/SQSTM1 started to increase 3 h after SPL treatment, reaching a maximum at 10 h and then gradually decreasing. Additionally, the protein levels of LC3-II and p62/SQSTM1 increased in a dose-dependent manner upon SPL treatment. (Fig. 5B). We investigated the effect of TLR2/4 on the increase of LC3-II and p62/SQSTM1 protein expression induced by SPL. The increase in SPL-mediated LC3-II protein expression was reduced by the inhibition of TLR2/4 induced by C29 and TAK-242. However, the increase in p62/SQSTM1 protein expression induced by SPL was only decreased by TAK-242-mediated TLR4 inhibition.

## Discussion

The immune system comprises both innate and adaptive immune responses [9]. Innate immunity recognizes and eliminates foreign pathogens in the early stages of infection, while adaptive immunity removes foreign pathogens through cytotoxic reactions and the secretion of antigen-specific antibodies [9]. Macrophages, one of the innate immune cells, play a crucial role in maintaining homeostasis by phagocytosing foreign pathogens and secreting immunostimulatory mediators (NO, iNOS, IL-1β, and TNF-α) to eliminate them from the body [10, 11]. In addition, activated macrophages provide antigen information to adaptive immune cells such as T cells and B cells, and the immunostimulatory mediators secreted by activated macrophages are reported to activate T cells and B cells [12]. Therefore, the activation of macrophages can enhance the functions of both the innate and adaptive immune systems in the body. Here, we observed that SPL increased the production of immunostimulatory factors such as NO, iNOS, IL-1β, and TNF-α and phagocytotic activity in RAW264.7 cells. These results demonstrate that SPL can have a positive impact on the activation of macrophages, suggesting its potential as a functional food ingredient that could enhance human immunity.

It is known that the immune response of the human body against invading foreign pathogens begins with innate immune cells such as macrophages recognizing foreign pathogens through pattern recognition receptors (PRRs)[13]. Toll-like receptors (TLRs), a type of macrophage PRRs, are essential for both innate and adaptive immune responses [14, 15]. Among TLRs, TLR2/4 are the most specialized PRRs for recognizing foreign pathogens [16]. In addition, when macrophages recognize foreign pathogens through TLR2/4, they are activated to produce immunostimulatory mediators [17]. In this study, we found that although TLR2 inhibition slightly downregulated the SPL-mediated production of immunostimulatory factors and activation of phagocytosis, it was observed that the increase in immunostimulatory mediators and phagocytic activity by SPL was significantly reduced by inhibition of TLR4. These results suggested that TLR4 is one of the major PRRs involved in SPL-induced macrophage activation.

MAPKs participating in TLR signaling are important in the human immune system. These MAPKs contribute to the production of immunostimulatory mediators by the activated macrophages [18, 19]. Of the MAPK constituents, ERK1/2, p38, JNK, p38, and ERK1/2 are important for the secretion of immunostimulatory mediators [20, 21]. In this study, we observed that the inhibition of p38 signaling suppressed SPL-mediated increase in the production of immunostimulatory mediators, and the inhibition of TLR2/4 blocked SPL-mediated phosphorylation of p38. These results suggest that SPL induces TLR2/4-dependent p38 activation.

Autophagy is closely related to the enhancement of innate immune responses because it activates the functions of macrophages such as antigen recognition, antigen presentation, and phagocytic activity [19]. LC3 is a marker used to investigate autophagosomes and autophagic activity, as it plays a role in recruiting foreign pathogens to the autophagosomes [22]. The expression of LC3-II protein indicates the activation of autophagy, as LC3-II is formed from LC3-I. Therefore, an increase in the expression of LC3-II protein indicates activation of autophagy [23]. p62/SQSTM1 is an important receptor that positively affects selective autophagy by delivering foreign pathogens to the autophagic pathway [24]. Thus, an increase in LC3-II and p62/SQSTM1 levels is a major indicator of autophagy. In macrophages, autophagy is activated when TLRs recognize foreign pathogens and form autophagosomes [25]. It has been reported that the activation of autophagy in macrophages via TLR4 enhances both innate and adaptive immune responses by improving antigen processing and presentation capabilities of macrophages [26]. In the present study, TLR2/4 inhibition downregulated the SPL-mediated increase in p62/SQSTM1 and LC3-II protein expression. These results suggest that SPL induces autophagy by stimulating TLR2/4 signaling.

Due to COVID-19 occurrence, interest in strengthening immunity has increased. While vaccine development is known to be the most optimal way to combat infectious diseases, strengthening the human immune system through diet is also considered an important defense strategy. A vegetable-based diet can strengthen the weakened immune system of the body that has been caused by Westernized dietary habits. In this study, SPL widely used as food in Korea induced macrophage activation through the p38 activation dependent on TLR2/4 and activated macrophage autophagy through TLR2/4 stimulation in RAW264.7 cells. These results indicate that SPL has immunostimulatory activity. Therefore, SPL could be used as a functional food agent to enhance the immune system.

## Figures and Tables

**Table 1:** The sequences of primers used in the amplification of the cDNA.

Primers	Sequences
iNOS	Forward 5'-ttgtgcatcgacctaggctggaa-3' Reverse 5'-gacctttcgcattagcatggaagc-3'
IL-1β	Forward 5'-ggcaggcagtatcactcatt-3' Reverse 5'-cccaaggccacaggtattt-3'
TNF-α	Forward 5'-tggaactggcagaagaggca-3' Reverse 5'-tgctcctccacttggtggtt-3'
GAPDH	Forward 5'-ggactgtggtcatgagcccttcca-3' Reverse 5'-actcacggcaaattcaacggcac-3'
